# ASD Diagnosis and Treatment Experiences Among Mexican Heritage Families

**DOI:** 10.1007/s10803-022-05512-3

**Published:** 2022-03-19

**Authors:** Shana R. Cohen, Jessica Miguel, Jessica Trejos

**Affiliations:** Department of Education Studies, University of California, 9500 Gilman Drive #0070, San Diego, CA 92093 USA

**Keywords:** ASD diagnosis, Autism spectrum disorder, Mexican-heritage families, Multiple case study design

## Abstract

To understand the ASD diagnosis and treatment pathways for US families, *N* = 38 Mexican-heritage mothers were interviewed about how and when they obtained an ASD diagnosis for their children. Most children (84%) were diagnosed between two and three years old. One-third of mothers reported receiving four to seven referrals before diagnosis. Mothers identified multiple diagnosis circumstances including two diagnoses and services offered before diagnosis. A multiple case study design documented the diagnosis and treatment experiences of four representative participants. As compared to previous studies that utilized a deficit lens to rationalize barriers to diagnosis and treatment (e.g., parents not knowledgeable about ASD), these findings revealed a complex understanding of how structural barriers (e.g., immigration status), initial diagnosis rejection among caregivers, and abrupt service cancellation complicated the diagnosis and treatment process. Implications identified suggestions to optimize and streamline ASD diagnosis and treatment pathways for Mexican heritage families.

**Supplementary Information:**

The online version contains supplementary material available at 10.1007/s10803-022-05512-3.

Quality early intervention (EI) (e.g., Naturalistic Developmental Behavioral Interventions) can improve outcomes for children with ASD and can result in considerable cost savings to service systems and families (Chasson et al., 2007; Muschkin et al., 2015). Children who participate in quality EI make gains in social, communicative and adaptive domains; these gains last over time (Anderson et al., 2014; Estes et al., 2015). In contrast, children with ASD who do not receive EI, or who receive it later in their development (after three-years old), have suboptimal outcomes (Clark et al., 2018).

To capitalize on this critical time of a child’s development, the Child Find and Referral mandate described in Part C of the Individuals with Disabilities Education Improvement Act (IDEIA) requires all states to locate, identify, and evaluate all children at risk for developmental delay (IDEIA, 2004). In California, these children are enrolled in Early Start, a program funded by the California Department of Developmental Services (CDDS). There are 21 Regional Centers across California that fund services and supports to individuals with disabilities. According to the Lanterman Act, regional centers are “the payor of last resort.” If a child needs Speech Therapy services, and that child has Medicaid, or if he attends a public school, the school, or the insurance company must pay for the service. The Regional Center cannot provide the funding. Age three is a critical transition period for many children receiving Regional Center Services like Early Start. When the child turns three and is not “substantially disabled” by three or more eligible conditions, then the Regional Center transfers funding responsibilities to the school district or the services are terminated (ACES, n.d.).

## Local and State Policies Impact ASD Diagnosis and Treatment for Immigrant Families

Few studies have examined the ASD diagnosis experiences of immigrant Latino families (Magaña et al., 2013; Zuckerman et al., 2013; Zuckerman et al., 2014; Zuckerman, Mattox, et al., 2014). Recognizing these experiences for immigrant families would allow policy makers to understand where barriers exist and develop policies to mitigate those barriers. We do know that for children from socioeconomically and culturally diverse backgrounds, biased societal structures have perpetuated racial and ethnic inequities, further limiting access to diagnostic and intervention services for ASD (Magaña et al., 2016; Mandell et al., 2002; Stahmer et al., 2019). In California, there exist spending discrepancies that impact socioeconomically and culturally diverse families’ access to ASD services. In 2013, the California Department of Developmental Services (CDDS) spent $9571 per Latino child, as compared to $11,480 per white child (Leigh et al., 2016). In response to CDDS’s significant spending disparities, California’s legislature passed Senate Bill 946, allowing Californians to claim ASD services under state health insurance policies (i.e., Medicaid) (Senate Bill, 946, 2011). Although studies show that this law provided access to ASD services for low-income families (LaClair et al., 2019), barriers still exist in service access with fewer autism and case management services provided to Black, Asian, Latino, and Native American eligible families in comparison to white families (Bilaver et al., 2020).

Similar discriminatory immigration policies have prohibited immigrant families from accessing timely diagnosis. After the passage of California anti-immigration policies (e.g., Proposition 187, 1994) that limited access by undocumented immigrants to social services, public health services, and education benefits, the number of ASD diagnoses waned among this population (Senate Bill 396, 2014). Once these policies were repealed in 1999, access to diagnostic services increased to former levels (Fountain & Bearman, 2011). Identifying as a Latino immigrant in California, at a particular point in history, predicted ASD risk (Fountain & Bearman, 2011). In our current political system, inequitable structural factors continue to display racial and ethnic inequities among undocumented immigrant families who rear a child with ASD (Luelmo et al., 2020).

## Healthcare Factors Impacting ASD Diagnosis and Treatment for Immigrant Families

Pediatricians and families may have differing perceptions about ASD and its symptoms, which could also impede timely diagnosis and treatment (Jegatheesan et al., 2010; Rivard et al., 2019). Jegatheesan et al. (2010) followed South Asian Muslim immigrant families for 1 and 1/2 years while parents described their experiences seeking diagnosis and treatment for their children with ASD. While parents consulted with a physician in their native language for initial visits, they were referred to European–American physicians for a diagnosis. Parents reported a lack of interpersonal skills and cultural awareness among the European-American physicians during diagnosis and post-diagnosis.

Other studies found a similar lack of cultural awareness and understanding of immigrant families and their children with ASD (Zuckerman et al., 2017). Lin et al. (2012) examined data from the National Survey of Children’s Health to understand how immigrant families who cared for children with developmental delays accessed medical and education services. Data from 413 households found that pediatricians took less time to understand and support immigrant families during the diagnosis process as compared to non-immigrant families (Lin et al., 2012). In another study, Zuckerman et al. (2013) found that of their sample of 267 California pediatricians, 11% offered ASD screening in English and Spanish. Even Spanish speaking practitioners reported difficulty assessing ASD risk for Spanish-speaking children. Finally, researchers examining the diagnosis practices of 38 pediatricians in the US-Mexico border community of El Paso, Texas found a low pediatrician to patient ratio; there were too few pediatricians available to adequately screen Latino children (Gonzalez, et al., 2015). Limited resources, insufficient time spent learning about the child and their family, and conflicting cultural beliefs about development and ASD symptoms between providers and families all inhibited timely access to ASD diagnosis and treatment for diverse families.

## Cultural Factors Shaping ASD Diagnosis and Treatment for Immigrant Families

Family language and cultural practices may contribute to when and how parents seek out ASD diagnosis and treatment (Blacher et al., 2014; Cohen & Miguel, 2018; Norbury & Sparks, 2013). One study examined immigrant families’ beliefs about the causes and symptoms of autism. Mexican immigrant families characterized their children as loving, affectionate, and empathetic (Cohen & Miguel, 2018). Authors interpreted these findings by discussing how families may wait to seek out services if their child exhibits affection and love to them. Families who do not see their child exhibiting atypical speech, or limited responses to social interactions, characteristics that have been used to diagnose autism (American Psychiatric Association, 2013), may not actively seek out a diagnosis. Families’ perceptions about their children’s character and skills likely shape how and when they seek out diagnosis and treatment (Cohen & Miguel, 2018).

Language factors have also been shown to limit access to ASD diagnosis and treatment (Zuckerman et al., 2017). Zuckerman and colleagues surveyed 352 English and Spanish speaking families caring for children with autism about the barriers in accessing ASD diagnosis and treatment services. They found that the participants who identified as Limited English Proficient experienced disproportionately more barriers in accessing diagnostic services than participants who were English proficient. A common barrier among this group was parents who reported limited knowledge of ASD (Zuckerman et al., 2017). These language barriers exist during ASD treatment as well. A recent study by Lim et al. (2020) used video modeling techniques to overcome language barriers between English speaking service providers and Spanish speaking families.

Few studies have examined the ASD diagnosis and treatment process for first and second generation Mexican mothers (Ferguson & Vigil, 2019; Lopez et al., 2018). Much of the research identifies common patterns showing that socioeconomically and culturally diverse children are diagnosed later, receive fewer ASD diagnoses and treatment, and are less likely to be diagnosed with ASD despite displaying similar ASD symptoms as white children (Blacher et al., 2014; Liptak et al., 2008; Thomas et al., 2012; Zuckerman et al., 2014; Zuckerman et al., 2014; Zuckerman et al., 2014). These studies used survey data, retrospective case finding, and focus group interviews. Few studies have used a multiple case study methodology to examine the complex contexts in which families seek out diagnosis and treatment (Luelmo et al., 2020). Understanding the contexts in which families obtain and receive ASD diagnosis and treatment is an important contribution to the field. Previous studies have shown that context impacts immigrant families' access to services (Fountain & Bearman, 2011). Current study findings will inform the development of individualized and accessible pathways to diagnosis and treatment for minoritized groups. In this study we examined ASD diagnosis and treatment timelines, the adults that may or may not have provided guidance and support throughout the process, and the contexts in which ASD diagnosis and treatment occurred for Mexican heritage^1^ families. To address the structural, language, and cultural inequities in access to timely ASD diagnosis, more studies are needed that examine the characteristics of ASD diagnosis pathways of socioeconomically and culturally diverse families. Current study findings will provide specific information about how services are accessed, detailed characteristics of the types of barriers that prevent timely access to diagnosis and treatment, and opportunities to adapt the process, eliminate barriers, and provide earlier access to appropriate services for other economically and culturally diverse families. In the current study, we examined the context in which diagnosis and treatment occurred for Mexican heritage families and their children with ASD. More specifically, we asked:

1. How do ASD diagnosis pathways for Mexican heritage families vary by age of diagnosis and number of referrals prior to diagnosis?
2. What are Mexican heritage families’ reported diagnosis circumstances and how do representative participants describe their experiences obtaining an ASD diagnosis and subsequent treatment services?

## Methods

### Research Design

A Case Study Methodology examined the pathways that led to ASD diagnosis and treatment for immigrant families. Case studies are used to enhance our knowledge of complex human experiences within a specific context (Stake, 2006). They provide detailed accounts of human behavior within the complex contexts in which participants live and learn (Merriam, 1998; Yin, 2017). The multiple case study approach—one case study that covers multiple cases—allows for increased validity, greater replicability and extension among individual cases, and a stronger base for theory building (Eisenhardt, 1991; Stake, 2006; Yin, 2017).

We implemented a multiple case study approach to develop a multi-faceted description of Mexican heritage families' varying pathways to ASD diagnosis and treatment. This approach also allows researchers to examine patterns, identify commonalities and draw a single set of cross-case conclusions (Eisenhardt, 1991; Stake, 2006; Yin, 2017).

## Participants

This study draws from an integrated methods study examining ASD beliefs, ASD diagnostic pathways, and treatment decisions made by Mexican heritage families who rear children with ASD (Cohen & Miguel, 2018). Families were recruited from two sources: (1) a private, non-profit regional center that provides services to individuals with developmental disabilities, including ASD; and (2) a medical clinic located in a border city between the US and Mexico, that provides ASD diagnosis services to immigrant families. Both institutions serve a large Mexican immigrant population. The regional center employs psychologists to conduct the Autism Diagnosis Observation Schedule (ADOS) and provide an ASD diagnosis (Lord et al., 2008). The medical clinic employs developmental pediatricians to diagnose ASD with medical screening tools, parent interviews, and child observation.

The institutions hand delivered or mailed out bilingual recruitment packets to eligible participants (i.e. Latino, Spanish speaking, ASD diagnosis) that included a letter from the respective institution describing the study and its independence from the services they receive. Packets also included a university recruitment letter describing the study, an opt-in card, and a stamped envelope with the university address to each eligible study participant. Interested parents completed and returned the opt-in cards. The second author called parents who returned the opt-in cards (97% mothers), determined eligibility and interest, and scheduled an interview. Researchers received 53 opt-in cards. Fifteen of these potential participants were not enrolled because they did not meet eligibility criteria, they did not return calls for enrollment (i.e. wrong number, or phone number was disconnected) or they were unable to schedule an initial meeting. The final sample included 38 caregivers of children with ASD (86.8% from the Regional Center; 13.2% from the medical clinic). Coincidentally all study participants were born in Mexico or had at least one parent who was born in Mexico.

### Data Collection Procedures

Researchers conducted home visits between August and November, 2016. During the visit, parents were asked to complete an online demographic survey and participate in a semi-structured interview, about their experiences obtaining an ASD diagnosis. Participants received $25 in remuneration. Some families reported rearing multiple children diagnosed with ASD. Researchers asked them to describe the diagnostic pathway for one target child, all participants independently selected to discuss their male child with ASD.

The first author developed the bilingual interview protocol and piloted it with other parents who cared for children with developmental disabilities. She conducted five interviews, while training the second author. Then, both authors conducted interviews primarily in Spanish, in a location convenient for the family (e.g., in families’ homes, local libraries or parks), based on participants’ availability. Before home visits, in an initial study phase, researchers developed rapport with participants through focus group interviews (Cohen & Miguel, 2018). When entering families’ homes we respectfully followed their rules for guests (e.g., removed shoes). Interviews ranged from 31 to 148 min (Mean = 64.58, SD = 20.99). Researchers verified the information from study participants by periodically summarizing the participant’s answers during the interview. Audio recorded interviews were transcribed verbatim and uploaded to Dedoose for analysis. Sample interview questions included: “Take me back to the time when you first received your child’s diagnosis. Please tell me the details about when your child was diagnosed, who did you speak to, what did you do, so that I can understand how you came to receive the services that you currently have.” Clarifying interview questions were added to fully understand each participant’s individual diagnosis experiences. All three authors shared a linguistic and cultural background with study participants.

### Data Analysis Procedures

There were four levels of analysis. First, researchers categorized all 38 participants based on child age at diagnosis and number of adult referrals before diagnosis, two disparity markers in access to diagnosis (Magaña et al., 2013; Mandell et al., 2009). To obtain the child’s age of diagnosis, researchers reviewed interview questions asking the mother to identify the age at which her child was diagnosed. To obtain the number of referrals before a confirmed ASD diagnosis researchers reviewed interview questions asking the mother to recall the details of who identified concerns about their child’s development. Referrals were defined as the mother reporting that another respected adult (e.g. a teacher, physician) or family member (e.g. grandmother, sister) in the community reported concerns about the child’s behavior or their development and may have suggested that the family seek out a diagnosis, or seek additional support for their child.

Second, to understand the context of ASD diagnosis circumstances, transcripts were coded for ASD diagnosis circumstances, or mothers’ reported experiences obtaining a diagnosis for their child. Diagnosis circumstances were coded when mothers were asked to describe the circumstances that led to their child’s diagnosis. Interviews were analyzed and coded using inductive coding methods (Miles et al., 2014). Researchers identified each participant’s diagnosis experiences, beginning at the mothers’ first reported developmental concern and ending upon the mothers' report of a diagnosis. This heuristic coding method revealed several diagnosis circumstances—parent-reported specific experiences that characterized the ASD diagnostic process. Authors individually read through one-third of the participants’ interviews. Then authors collaboratively categorized, combined, and organized diagnosis circumstances to account for individual coder descriptions. Any discrepancies were discussed during the coding meetings where coding definitions were revised and finalized. Researchers finalized the coding definitions of all diagnosis circumstances and collaboratively re-coded all participant interviews based on the final coding definitions. Participants’ diagnosis circumstances were double coded if necessary.

To further understand the complex ASD diagnostic pathways of participants, in a third level of analysis, researchers identified four representative mothers that reflected the diversity and complexity of diagnosis circumstances of all participants (Eisenhardt, 1989). Using Stake’s case study selection guidelines (2006), researchers developed operational criteria to identify representative study participants that illustrated the variability of the sample. Participants were selected using three ASD diagnosis characteristics (Palinkas et al., 2015); (1) the diagnosis circumstances pertaining to that participant; (2) the child’s age at ASD diagnosis, and (3) the number of referrals before diagnosis. To identify representative cases, each author reviewed one-third of the transcripts and the corresponding diagnosis circumstances for each participant. Then researchers met and summarized each study participant’s diagnosis characteristics. Researchers collectively identified four participants that represented the range of experiences across the three operational criteria. The four case study participants represented: (1) eight of the 11 diagnosis circumstances; (2) child diagnosis ages ranging from 1 year old to 7 years old; and (3) number of referrals before diagnosis ranging from two to five. This case study selection criteria represents the diversity of experiences defined by the three diagnostic characteristics discussed above and is aligned with common sampling techniques; cases reflect a broad range of experiences and enhance generalizability (Eisenhardt, 1989).

### Narrative Analysis

In a fourth level of analysis, researchers reviewed the four representative participants' descriptions of the events that led up to their child’s ASD diagnosis using *dialogic analysis*—researchers’ evaluation of the dialogue between the participant and the interviewer to examine participants’ concerns, observations, and experiences (Matsuov et al., 2019; Riessman, 2008). Researchers aimed to capture the detailed sequence of events as the mother reported them from the first episode of developmental concern to the final episode of ASD diagnosis (Riessman, 2008). Researchers examined the contexts in which decisions were made and the other individuals (i.e. adults and/or children) that were reported as influencing the mothers’ decision making process. Researchers were also interested in understanding how each mother’s sense of agency and her advocacy skills shaped the ASD diagnosis trajectory (Riessman, 2008). From mothers’ reports of the diagnosis timeline for their child, researchers generated a referral narrative—a descriptive summary of the participant’s ASD diagnosis pathway (Gabel & Kotel, 2018). To maintain data integrity, researchers used participants’ words to describe major milestones of each family’s diagnostic pathway and the third author read the referral narratives to verify data accuracy.

## Results

### Demographics

The larger sample included 38 parent–child dyads that were recruited for this study. Children ranged from 3–15 years of age (*M* = 5.89) and on average were diagnosed at 3.15 years of age with an average of three referrals prior to receiving an ASD diagnosis. All child participants were male and six out of the thirty eight held a second diagnosis (e.g. Neurofibromatosis, intellectual disability, hydrocephalus, mental retardation, speech delay, attention deficit disorder, epilepsy, and hyperactivity). All of the child participants were receiving some type of early intervention, the majority received services at school (26%) and 18% received less than 20 hours(h) a week of one on one home intervention (i.e. Applied Behavioral Analysis). All students were also enrolled in school with the majority (50%) attending a general education school and classroom, and about one-third of the children (37%) attending a special education classroom in a traditional public school (See Table 1). Mothers ranged in age from 24 to 53 years (*M* = 36.21), the majority (74%) were married or living with a partner, born in Mexico (82%) and reported speaking primarily Spanish in the home (87%). Parents reported 11th grade or less (31%), Some College (26%), or a Bachelor’s degree or higher (21%). Many of the mothers were unemployed at the time of data collection (74%) and were making under $35,000 a year (61%). Key information related to each case study participant is provided in the narrative analysis (See Table 2).

**Table 1:** Demographics for mother participants (N = 38)

Mothers	Mean (SD)	Range	Frequency	%
Age	36.21 (5.92)	24–53		
Married or living with a partner			28	73.7
Single			10	26.3
Born in Mexico^a^			31	81.6
Years living in the US	13.50 (7.21)	2–36		
Born in the United States^b^			7	18.4
Spanish primarily spoken at home			33	86.8
English primarily spoken at home			5	13.2
Level of education				
11th Grade or less			12	31.5
High school/GED			6	15.8
Some college			10	26.3
Bachelor’s degree or higher			8	21.0
Choose not to answer			2	5.3
Currently working			10	26.3
Unemployed			28	73.7
Household income				
Under 15,000			6	15.8
15,001–25,000			10	26.3
25,001–35,000			7	18.4
35,001–50,000			7	18.4
Choose not to answer			8	21.1

^a^All adult participants reported having a mother who was born in Mexico and 97% (N = 37) reported having a father born in Mexico

^b^The seven participants self-reported their ethnicity as Hispanic and all seven had parents born in Mexico

**Table 2:** Demographics for child participants (N = 38)

Children	Mean (*SD*)	Range	Frequency	%
Age	5.89 (2.26)	3–15		
Age of ASD diagnosis	3.15 (1.74)	1–11		
Number of referrals	3.08 (1.65)	1–6		
Male			38	100
ASD diagnosis			32	84.2
ASD & other diagnosis^a^			6	15.8
Current enrollment in early intervention^b^				
Center based intervention^c^			1	1.3
1:1 Home intervention (> 20 h a week)			2	2.6
1:1 Home intervention (< 20 h a week)			14	18.4
Intervention based in school			20	26.3
Other (e.g. Therapy)			2	2.6
Enrolled in school				
Typical class in regular school			14	36.8
Special day class in regular school			19	50
Separate school			2	5.3
Home school			2	5.3
Receiving special services in school^d^			32	84.2
Receiving services at a Clinic				
Therapy^e^			8	21.1
Pediatric^f^			4	10.5
Receiving services outside of a school or clinic			15	39.5

^a^Neurofibromatosis, intellectual disability, hydrocephalus, mental retardation. speech delay, attention deficit disorder, epilepsy, and hyperactivity

^b^Participants may list up to two Early Intervention services (as defined under Part C of IDEIA), 26 out of 38 participants are enrolled in at least one intervention service

^c^The child attends a group based intervention program at a center

^d^e.g. 1:1 aide and speech therapy

^e^e.g. Language therapy, occupational therapy, behavioral therapy, speech therapy, and physical therapy

^f^e.g. Pediatric Clinic, Clinic, pediatrician, developmental pediatrician

### Patterns of ASD Diagnosis

**Fig. 1:**
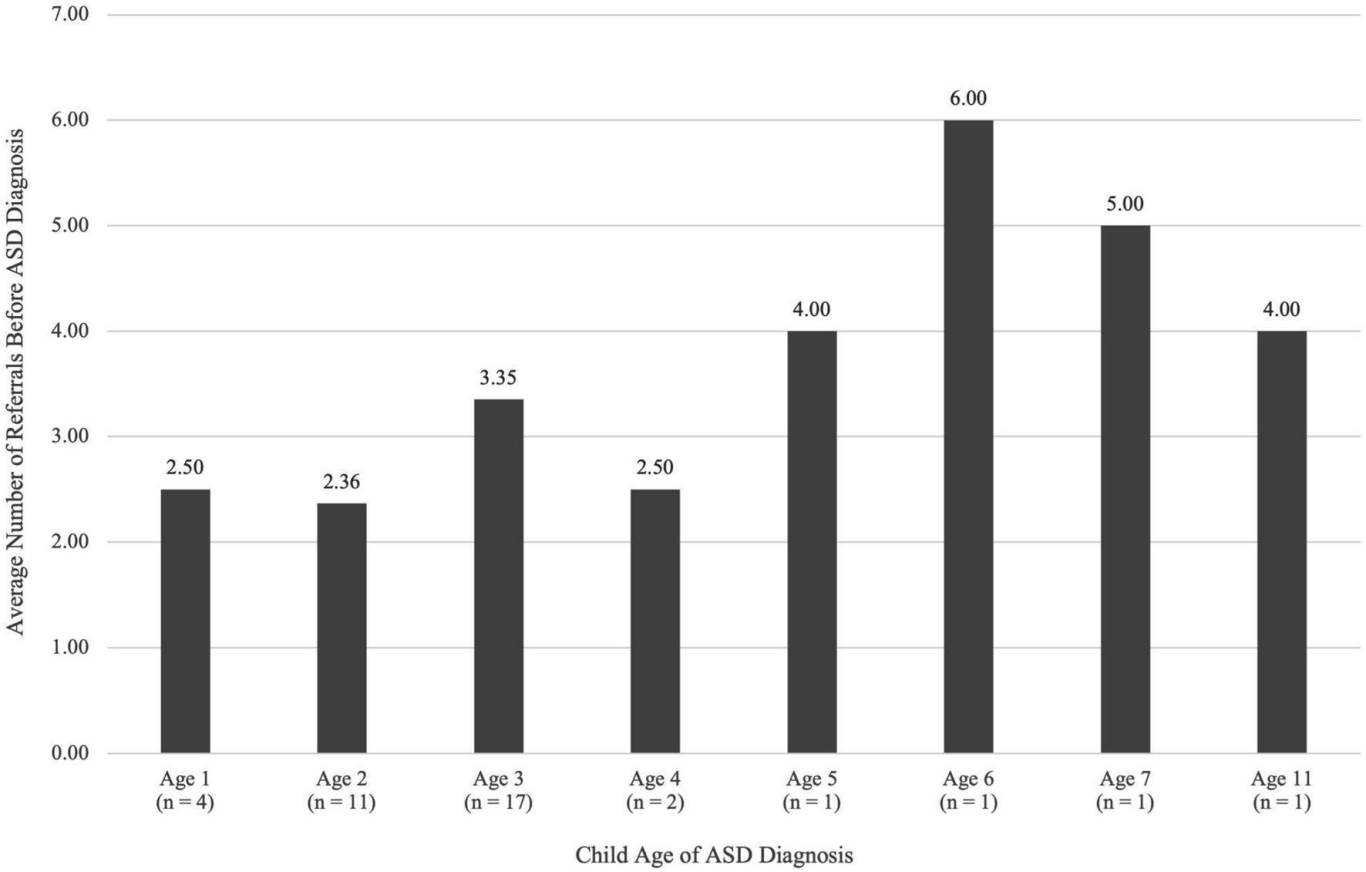
Child’s age of ASD diagnosis by the average number of referrals before ASD diagnosis (N = 38)

The first research question examined how the common ASD diagnosis pathways for 38 Mexican heritage families varied in relation to the child’s age of diagnosis and average number of referrals before diagnosis (See Fig. 1). Children who were diagnosed at age one (n = 4) had an average of 2.5 referrals and those diagnosed at age two (n = 11) had an average of 2.36 referrals. Children diagnosed at age three (n = 17) had an average of 3.35 referrals and those diagnosed at age four (n = 2) had an average of 2.50 referrals. Four children were diagnosed with ASD over the age of four, one child was diagnosed at age five with four referrals, another child had six referrals and was diagnosed at age six, a third child was diagnosed at age seven with five referrals, and a fourth child had four referrals with a diagnosis at age 11. A majority of child participants, 74% (n = 28) were diagnosed between two and three years old. Child participants received one to seven referrals before diagnosis. The largest number of children (45%; n = 17) received an average of 3.35 referrals before diagnosis, and a small, but older, portion of child participants (10%, n = 4) had more than four referrals prior to ASD diagnosis.

To contextualize these findings, we also examined the diagnosis circumstances, or the specific structural factors that characterized families’ diagnosis trajectories (See Table 3). The most frequent diagnosis circumstances included 11 parents who reported receiving services before formal diagnosis and 14 parents who reported living in a rural area with limited access to diagnosis and treatment. Many parents reported having to drive over 200 miles to the closest metropolitan city to obtain diagnostic services from the children’s hospital. Participants infrequently endorsed “Experience with ASD Diagnosis” in which parents reported having experienced the diagnosis process with an older child. Parents also infrequently endorsed “Little Knowledge,” in which parents reported knowing little information about ASD. Over half of the participants (55%; N = 21) reported experiencing two or three diagnosis circumstances, whereas 40% (N = 15) reported experiencing one or fewer diagnosis circumstances. Two families reported four or more diagnosis circumstances.

**Table 3:** Parents’ reported ASD diagnosis circumstances

Code label	Code definition	Frequency**
Little knowledge	Parents reported no developmental warning signs in their child, having no relatives with ASD, or little or no knowledge of ASD	1
Unclear path	Parents reported a non-linear or unclear path to receiving a diagnosis	4
Experience with ASD diagnosis	Parents reported having already experienced the referral process with an older child diagnosed with ASD	1
Mexico diagnosis*	Parents reported receiving a diagnosis in Mexico	6
Services before diagnosis*	Parent reported that child received ASD intervention treatment before receiving a formal diagnosis	11
Missed early diagnosis*	Parents reported that the child could have received a formal diagnosis but did not due to a doctor’s missed diagnosis, or a parent/grandparent not documenting the child’s developmental milestones	8
Two diagnoses*	Parents reported receiving a formal diagnosis from one health care provider and then another formal diagnosis from another health care provider	8
Developmental-behavioral pediatrician*	Parents reported developing a positive rapport with a particular Developmental- Behavioral Pediatrician during diagnosis	8
Little professional support*	Parents reported receiving little or no support during the diagnostic process	6
Rural county*	Parent reported living in a rural area with limited access to health and education services	14
Efficient, positive diagnostic pathway*	Parents reported a positive experience of the referral process	3

^*^Participants featured in the narrative case study analysis. Circumstance for Elena: Positive case. Circumstances for Emilia: Mexico diagnosis, Two diagnoses. Circumstances for Marcela: Services before diagnosis, Rural county. Circumstances for Graciela: Missed early diagnosis, Developmental Behavioral Pediatrician, Little professional support. **Participants’ referral circumstances were double coded if necessary

### Referral Narratives

To examine how the diagnosis pathways differed for representative participants, referral narratives below exemplify the diverse but representative ASD diagnosis experiences of our participants. Mothers’ immigration age ranged from 16 to 38 years old. All four mothers spoke Spanish with their children and were immigrants from Mexico. The following narratives translate direct quotes to English. Please see Supplementary Materials for Spanish quotes.

#### Elena^2^

Elena was 39 years-old, married, unemployed and completed the 11th grade. Her husband was also an immigrant from Mexico who worked at a restaurant. The family lived in urban Southern California and reported an annual income range of $15,001–$25,000.

Elena had one 5-year-old child, Joaquin, diagnosed with ASD at age two and a half after two referrals. Elena and the pediatrician noticed that prior to age two, Joaquin was not talking or vocalizing. He was referred to a clinic at the local Children’s hospital. The pediatrician would not diagnose him, but he referred him to the Regional Center. She explains:

The person who saw him there, [my child] did not want to interact with the people, he just stayed away, he didn’t want them to talk to him, all he did was play. Therefore [the clinician] told me, ‘You know what? I cannot give you a diagnosis, but I am going to refer him to the Regional Center.

At 2 years old, the Regional Center assessed him for vision and hearing and provided ABA, Speech, and Occupational services prior to the formal diagnosis. Elena was content with the services Joaquin received from the Regional Center, less than 20 h of ABA per week, “I have had a very good experience with them, they have helped me a lot. They have sent me therapies for everything.” At age two and a half, after receiving another vision and hearing test, the Regional Center psychologist diagnosed him. The psychologist added additional therapy hours (20 h per week, 4 h per day) and explained that home based behavioral therapy was set to end when Joaquin turned three years old. At that point Joaquin would receive Speech and Occupational therapy from school. No further explanation was provided as to why services would end at three years old.

Elena described a positive experience with Joaquin’s school, explaining that it was the most effective in helping Joaquin meet his goals. Teachers often provided support for academic and socio-emotional skills. She noted that Joaquin, at 5 years old, expressed himself well:

...in three years I have seen a big difference...but from three to four when he started in special education, that's when he grew the most; the biggest gains he had were in speech, his ability to express himself, to comprehend, to understand things, but it was the school that helped me.

Elena experienced timely diagnosis and treatment; she believed the Regional Center and the school contributed to her positive experience.

#### Emilia

Emilia is a high school graduate and single mother of three boys (15, 9 and 6-years-old), all of whom were diagnosed with ASD. Emilia was 42 years old and unemployed. Her reported annual family household income ranged from $15,001 to $25,000. Her oldest child, Eric, had the most severe ASD symptoms, with a comorbid diagnosis of attention deficit and hyperactivity disorder. His current, and second, ASD diagnosis occurred at 7 years old after five referrals. Eric was homeschooled by his mother, while receiving intervention services and Occupational Therapy at a local clinic. Emilia’s narrative highlights several referral circumstances, including an initial diagnosis in Mexico and a second diagnosis in the US. Emilia described how her referral narrative was shaped by her perceptions about child development and her family members’ perceptions of typical development.

The path to the first diagnosis began in Mexico. Emilia’s mother noticed Eric (age four) engaging in unusually aggressive behavior and brought it to her attention. Emilia described her mother’s perceptions:

‘I raised you all and [you] never had these tantrums, the boy is very strong willed and I do not think this is normal.’ So she had the experience to tell me: ‘I had babies, I had all four of you and none of you were like this, so something is up.’

Eric’s grandmother took him to a psychologist in Tijuana who noticed ASD symptoms and encouraged them to seek an ASD diagnosis. However, Emilia did not believe her child was developing abnormally and she did not pursue a diagnosis.

Upon school entry in Mexico (age five), Eric’s teachers reported concerns about Eric’s atypical behavior, including aggression and dysregulation:

And he was throwing a lot of tantrums, and I think that when he was throwing a tantrum, they tried to restrain him, he would hit the people that were trying to restrain him. That’s where they said: ‘You are going to have to find help.’ And that is where they recommended me to Pelas.^3^..where they diagnosed him....

Eric’s teachers encouraged Emilia to seek out behavioral support for Eric and obtain a diagnosis. The principal recommended Eric see a neurologist. The neurologist would not diagnose him but he did give Eric medication to control his aggressive behavior. One year passed. When Eric turned six, the school principal, still concerned about his behavior, referred Eric to a diagnostic center in Tijuana. The psychologist at the center in Mexico gave Eric his first diagnosis at 6 years old.

At age seven, Eric’s family immigrated to the US and Eric received his second diagnosis from the Regional Center. His school provided Eric with a few hours of ASD intervention but Emilia did not seek out additional therapy because she could not access MediCal as an undocumented immigrant and she was unable to pay for extra therapy. The family’s undocumented status further compounded the barriers to services:

We still were not in a legal situation, we were not immigrated. Thank God now we are... That is where the situation started, because we were undocumented. They couldn’t help him because we didn’t have MediCal, and I couldn’t pay for it. All I could do-- they told me: ‘The only thing you can do is what the school gives you.’

Upon gaining legal status, Emilia gained access to MediCal and obtained behavior therapy for Eric, now 12 years old. She met with a lawyer who helped Eric move from general education to special education so that he could receive speech and behavior therapy at school.

Emilia reflected upon how obtaining services for Eric shaped the referral trajectories for her younger children, also diagnosed with autism. Her early experiences motivated her to seek out early diagnosis and treatment:

So I said: ‘How did I not notice all of these details. If only I had given him help since he was younger.’ I began giving Salvador [youngest child] help before he turned one [year old], I began to push. ‘It’s because I want them to begin diagnosing him, I want them to start knocking on doors, Hospitals, Clinics. I want them to put me on a waiting list because I want him to be detected on time because I don’t want my son to go through what Eric [eldest child] did.’ From not believing [initially], now I was getting ahead of it…

Emilia’s narrative illustrates a complex process of obtaining services for Eric, questioning her own perceptions of typical child development, navigating her undocumented status upon immigrating to the US, and reflecting on her advocacy efforts to obtain service for her younger children with ASD.

#### Marcela

Marcela is a 43 year-old mother of six children, the youngest two, one male and one female, have a diagnosis. At the time of the study, Marcela was unemployed and had some college or vocational training. Previously divorced, her current husband was born in the US and was unemployed. Marcela reported an annual family household income range of $25,001–$30,000. Marcela described how her 6 year-old son, Isaiah, was diagnosed at age three, with severe ASD after five referrals. Marcela and her children initially lived in Mexico. When Isaiah turned 1 year, 8 months and his sister was three years old, she divorced her first husband and immigrated to the US. Upon arrival, she immediately sought a referral for her three-year old daughter based on previous concerns. Her daughter’s referral led Marcela to notice signs of ASD in her younger son:

So, once they [The Regional Center] diagnosed my daughter, I began to notice Isaiah. I commented here that I would like them to see Isaiah, because I saw something in him too that wasn't normal, he did not respond when I talked to him, he didn't turn, he didn't babble at all… He was very calm, he could lay down and do nothing, not even cry, nothing. For me it wasn't normal.

The Regional Center acknowledged Marcela’s concerns and offered Isaiah a spot in Early Start, a program that adheres to IDEA Part C requirements, for children who may be at risk for developmental delay but not yet diagnosed. The Regional Center psychologist would not diagnose Isaiah until he turned three years old. Upon receiving the diagnosis they immediately began weekly ABA therapy at home:

...he attended that program until three years old, and at three years old they placed him with the other agencies, like Molova… After the diagnosis, it wasn't a lot, because it was only orienting myself to his ABA therapies. That’s it.

Isaiah received 10 h per week of ABA therapy for three years. When Isaiah turned six, the Regional Center abruptly canceled services, without providing a reason to Marcela. However, Marcela believes that this service cancelation caused her son to regress in his communication skills and it has drastically affected his development:

I think that ABA helped Isaiah, Molova [Provider] helped him... a lot. He was already learning how to try to communicate with his pets, and now he has lost that, but he was trying… I mean, it was a big development because he was beginning to understand, ‘I want something, I have to demonstrate something.’ So now he has lost that. That was the little bit of communication he had.

Marcela believed that the additional ABA services were instrumental in supporting Isaiah’s communication. Marcela described her attempts to restore services but she felt powerless, frustrated, and anxious:

I have come to ask: ‘And when?’, ‘No, he is on the waiting list’, ‘Okay.’ Well, I am tired of asking them, but also I can’t just sit and wait, because it could not arrive. So right now I’m like: ‘What do I do? What do I do? He is not talking, not advancing.’ It is difficult.

Marcela’s narrative illustrates the irregular, non linear, process between diagnosis and treatment. Marcela obtained EI services before receiving a formal diagnosis in an effort to mitigate developmental risk, yet subsequent ABA treatment that supported his communication was abruptly canceled without explanation. At six years old Isaiah was attending a special day classroom, still unable to communicate with words.

#### Graciela

Graciela was a 44 year-old self-employed, single mother of four with no formal schooling and a reported annual family income under $15,000. Graciela’s son, Aron, was 6 years old and was diagnosed with ASD, hydrocephalus, and mental retardation at age one after two referrals. Graciela’s narrative highlights several referral circumstances, including limited professional support and a series of challenging transitions. She mentioned feeling guilty because she believed that Aron’s diagnosis stemmed from her struggle with alcoholism during pregnancy.

Aron received an initial diagnosis from a home visiting nurse at a local health clinic before age one. The nurse noticed symptoms consistent with ASD (e.g., biting, involuntarily hitting himself) and expressed her concerns to Graciela.

And I had already met that nurse many years ago. And when she saw my child hit himself, biting, and only [playing] with a jar, she said: ‘Don't be upset, but I think that your child has autism.’ I told her: ‘I don't believe that,” and I asked that they switch nurses because she told me that. And then, with the evaluations already, with all the studies, there was one where we had to go to Children’s Hospital three times a week, four times a week. Afterwards, I wasn’t as upset, but when they told me for the first time, I was upset. I got really upset and they changed the nurse that was coming to see my child and work with me.

Graciela had difficulty trusting the nurse’s opinion, yet she still took Aron to the local clinic to get evaluated. He was officially diagnosed by a developmental pediatrician and referred to the Regional Center, where a psychologist evaluated and diagnosed him again. After that diagnosis, Graciela began interpreting Aron’s behavior such as his difficulty with speech and basic tasks (e.g. going to the restroom, putting on clothing, brushing teeth) as consistent with an ASD diagnosis. Aron received Behavioral, Speech, and Occupational Therapy.

When Aron turned three years old, the Regional Center canceled all services claiming that the school district was now responsible for providing services:

It was now, ‘Okay. He is three years old, he is going to Head Start.’ Then Head Start told me: ‘No, no, no.’ They had him going from one place to another. ‘It’s that now it’s the responsibility of the district because it’s different now.’ The Regional Center did not want to see him. And I fought a lot until they re-evaluated him, they gave him another evaluation.

The school district finally agreed to re-evaluate Aron. As a result, Aron received an Individualized Education Plan (IEP) including Speech services and ABA therapy every week.

When Aron turned five, he entered Transitional Kindergarten. This transition prompted the school district to request a third evaluation that yielded a different diagnosis (Speech Impairment). He did not qualify for the same services he received with the ASD diagnosis through the Regional Center. Ultimately, Aron received Speech Therapy and a one-on-one aide in his special day class. Graciela initially did not interpret Aron’s behavior as consistent with ASD. Throughout multiple diagnoses, she reported frustration with constant transitions between service providers and the school’s decision to cancel services. She also advocated for Aron to receive the service she believed he needed.

## Discussion

Previous research has relied on large scale studies of Latino families answering brief surveys about their ASD diagnosis experiences to understand how Latino families participated in the ASD diagnosis process (Liptak et al., 2008). Previous studies also used surveys to identify barriers to diagnosis and treatment (Zuckerman et al., 2017). These studies framed barriers to treatment for Latino families from a deficit lens (e.g., parents are not knowledgeable about ASD, parents are too stressed to seek treatment) (Zuckerman et al., 2017). Our study provides a more nuanced understanding of the contexts in which these families engaged with service providers. For example, our study families identified more complex barriers to seeking out diagnosis and treatment than has been documented in previous studies. Moving beyond structural characteristics, families encountered personal challenges resulting in delayed acknowledgement of ASD, challenges related to their documentation status, and abrupt service cancellation. These challenges required participants to engage in strong advocacy efforts with multiple providers over many years. Findings will allow policymakers and practitioners to identify adaptations to policies and practices that integrate the trusted people and resources that some Mexican heritage families rely on to access an ASD diagnosis and treatment.

From this study we learned that although mothers' reported complex diagnosis circumstances that could have jeopardized families’ access to timely diagnosis and treatment, many mothers still reported a timely diagnosis. In reference to Research Question One, 74% of participants (*n* = 28) reported their child was diagnosed between two and three years old. Recent studies have identified the mean age of diagnosis to be between four and five years old for white, and Black middle class families and higher for Latino families (Baio, et al., 2018; van’t Hof et al., 2021). Previous studies have also identified median ASD diagnosis rates between two and four years old (Christensen et al., 2018; Christensen et al., 2016). Studies examining a child’s age at diagnosis identified disparities in ASD diagnosis for Latino children as compared to non-Latino children (Wiggins et al., 2020; Zuckerman et al., 2017). Findings show that Latino children are less frequently diagnosed with ASD (Christensen et al., 2016), rarely diagnosed under the age of four (Christensen et al., 2016), and are diagnosed later than non-Latino white children (Mandell et al., 2009). Fewer reports did not find ASD diagnosis disparities between Latino and non-Latino children (Ferguson & Vigil, 2019). Researchers attributed this finding to parents’ increased awareness and knowledge of ASD symptoms, or fluency in English (Christenson et al., 2018; Ferguson & Vigil, 2019). Studies of other minoritized groups found that age of diagnosis was correlated with the time children started school (Hall-Lande et al., 2018). In our study, most parents reported a timely diagnosis. The Child Find and Referral state policies carried out by healthcare workers and Regional Center providers may have afforded study participants better access to early ASD diagnosis and treatment (§52040, 2017). Some studies have also shown that new policies allowing for Medicaid eligible families to access treatment at no cost have allowed economically and culturally diverse families to access timely diagnosis and treatment (LaClair et al., 2019).

Findings from Research Question One also showed that 21 participants (55%) reported between three and seven referrals before receiving a diagnosis. A family who reports having to seek out two or more diagnoses before obtaining the appropriate services may be encountering barriers due to racism or clinician bias. Race-based differences in obtaining an ASD diagnosis have also been acknowledged in previous studies (Mandell et al., 2002; Ratto et al., 2016). Black children who received MediCal required three times as many visits to health care providers for a diagnosis, as compared to white children (Mandell et al., 2002). Parents in our study who interacted with multiple health care professionals before receiving a diagnosis reported having to engage in strong advocacy efforts. Zuckerman et al. (2015) suggests that proactive provider responses (e.g., developmental screenings) to parents' concerns of ASD symptomatology can support parents’ advocacy efforts. Lopez et al. (2020) have also argued for practitioners to engage in cultural humility to learn about how Latino families perceive ASD symptoms and connect families to culturally relevant resources in their local communities (Lopez et al., 2020). Widely available ASD screening tools should also be used to detect ASD early– at the child’s 1 year well baby visit (Pierce et al., 2011; Zwaigenbaum et al., 2015). In fact, researchers have identified specific screening tools that are robust indicators of ASD among Latino children that can more quickly screen for ASD in this specific population (Linares-Orama et al., 2019). Other studies noted a promising multi-stage ASD screening and evaluation tool that was effective among children from diverse backgrounds and those receiving public assistance (Eisenhower et al., 2021).

In reference to Research Question Two describing families’ diagnosis circumstances, almost 30% of participants reported receiving ASD intervention before diagnosis. This is not surprising given the increased awareness of ASD among families and physicians (Monteiro et al., 2016). There could be occasions when a diagnosis is not clear, but the child would benefit from early intervention services. In fact, Child Find and Referral state policies encourage clinicians to “find” and enroll children in intervention quickly given the abundance of evidence showing that early intervention optimizes child outcomes (Estes et al., 2015). Study participants also reported that many clinicians would not make a formal diagnosis until the child turned three years old. Children can be diagnosed much earlier than three years old (Pierce et al., 2019). Proactive, equitable healthcare provider responses and culturally appropriate, efficacious screening tools could stabilize diagnosis rates by race and ethnicity.

From Research Question Two, representative families reported how diagnosis circumstances (structural factors) and varying perspectives about development shaped the time to ASD diagnosis and treatment. Contrary to other studies showing that pediatricians hesitate to provide an ASD diagnosis (Crais et al., 2014), Elena described a positive experience. She had a responsive pediatrician who immediately acknowledged her concerns and supported her advocacy efforts. Elena also described improvements in Joaquin’s communication and comprehension skills. In previous studies, parents’ perceptions of developmental outcomes have shaped their priorities for their child’s development (Cohen & Miguel, 2018; DuBay et al., 2017). Dubay et al. (2017) found that Latino parents (as compared to white parents) were more likely to prioritize communication for daily life and personal safety. Elena’s satisfaction with Joaquin’s development may have contributed to her positive outlook about her son’s diagnosis.

Emilia and Graciela described complex referral narratives shaped by structural factors and personal beliefs about development that prevented an early acceptance of the diagnosis. For parents who emotionally struggle to acknowledge a diagnosis, children may be at risk for less intensive and lower quality parent–child interactions (Wachtel & Carter, 2008). Timing of acceptance is important as early access to quality interventions are crucial for optimal development. Once Emilia and Graciela accepted the diagnosis, they each immediately engaged in advocacy efforts to obtain services. Emilia found a diagnosis in Mexico and in the US with limited resources. Emilia’s ability to obtain a diagnosis in Mexico is important. Previous studies have found that it is difficult to obtain a timely diagnosis in Mexico due to families who lack access to healthcare and medical insurance, and doctors who lack medical training (Bravo Oro et al., 2014; Márquez-Caraveo & Albores-Gallo, 2011). After becoming a naturalized citizen in the US, and recognizing Eric’s diagnosis, Emilia immediately advocated for additional services. Parental advocacy programs have supported mothers’ initial frustration upon receiving a diagnosis, and later feelings of self-worth, given the perseverance required for effective advocacy (Boshoff et al., 2016; DePape & Lindsay, 2015). Researchers have begun assessing the efficacy of formal parent interventions to promote advocacy and empowerment among Latino families who have children with ASD (Burke et al., 2016; Magaña et al., 2017).

For Marcela and her family, the diagnosis process was not linear. Isaiah received services before a formal diagnosis, as many families in our sample reported. After three years of consistent, effective treatment, services were abruptly canceled without explanation, likely because Isaiah, age six, was eligible for services through his local school district. It is also possible that services were abruptly canceled because of limited service availability in her rural community. Recent studies show that families living in rural communities have more barriers to services than those living in urban communities (Burke, 2017; Murphy & Ruble, 2012; Zhang et al., 2017).

## Implications, Limitations, and Conclusions

Families’ complex and variable ASD diagnosis and referral experiences suggest that the current healthcare system lacks a standardized approach for obtaining a diagnosis. Local, state and regional healthcare systems must engage in systematic efforts to standardize and effectively communicate diagnosis procedures. Targeted outreach campaigns with clear and simple pathways to diagnosis that mitigate barriers and elevate parents’ sense of empowerment should be developed and advertised across the state and in local (and rural) communities. Elena’s story of a positive referral experience demonstrates how the level of satisfaction can be bolstered by being listened to when noting developmental concerns. Primary care providers should engage in equitable, efficient healthcare practices that acknowledge parents’ complex feelings about their child’s development while also engaging directly with families, and transparently communicating the pathway to diagnosis. Service providers should actively follow up with families who have experienced abrupt service cancellation, discuss the child’s continued needs, and collaborate with families and schools to develop a culturally relevant plan for continued support. Providers should also engage in transdisciplinary, interprofessional collaborations in which providers from different disciplines and families develop unified treatment plans that support the needs of the child and the educational goals of the family (Bowman et al., 2021).

Structural factors (e.g., undocumented status) also shaped families’ experiences in obtaining an early ASD diagnosis. Some of our participants refrained from advocating for their children because of their undocumented status. The current anti-immigrant sentiment and resulting policies that criminalize immigrants’ experiences and disenfranchise immigrant communities have been linked to reduced access to mental health services (Bozorgmehr & Jahn, 2019; Ortega et al., 2018). Healthcare and immigration policies must be redesigned to strengthen and empower caregivers, regardless of immigrant status, to access the necessary health and human services to keep their families healthy and developing optimally. In US states that border other countries, policy makers from both countries should work together to align health and human service policies so that caregivers do not need to engage in aggressive advocacy efforts to obtain a diagnosis both in the families’ home country and then again in the host country. Previous studies have shown that the US, Canada, and Mexico show a large degree of agreement and convergence on these types of social policies but more is needed (Barnes, 2011).

Families also play a key role in ASD diagnosis. As evidenced by Emilia’s narrative, it was the child’s grandmother that first noted behavioral and developmental concerns which led to an initial doctor’s visit for ASD diagnosis. Research demonstrates that grandparents and extended family can play a pivotal role in autistic children’s lives, providing caregiving to the child and emotional support to parents that can strengthen the child’s environment (Gorlin, 2019; Prendeville & Kinsella, 2019). Therefore, interventionists and care providers should be aware and help parents navigate the integration of extended family into their daily routines. What is more, both Emilia and Marcela had multiple autstic children, as noted from their narratives. They described how the diagnosis of one child helped them obtain a diagnosis for their younger children, and that knowledge of the system was used to help them obtain more services for their other child(ren). This is important to note given research suggesting a lack of awareness of ASD and its characteristics among Latino families (Zuckerman et al., 2015). Latino families with autistic children can provide guidance to other parents with autistic children. Other studies have shown that promotoras, or more experienced families, help support families with autistic children (Magana et al., 2017).

A limitation of this study is that study families were already connected to the Regional Center service system. The study did not include families who have not yet begun the diagnosis process, or families that may have been overlooked by Child Find and Referral state policies. Given the fact that there are over two million undocumented immigrants residing in California, and around 78% are from Latin America (Hayes & Hill, 2017), there are likely many families who have been overlooked and who have children at risk for ASD. Future studies should examine diagnosis pathways of immigrant families who have yet to obtain support from state funded programs or school districts and who may live in rural communities. Pediatricians who work in border communities should utilize evidence-based screening tools specifically developed for Latino families to carefully screen their patients (Linares-Orama et al., 2019). Social workers and early educators should be trained to notice the signs of ASD and the names and up-to-date contact information of specific ASD diagnosis services to refer families in their local communities (Lopez et al., 2020).

Another limitation is that study participants were all mother-son dyads. The voluntary nature of our recruitment procedures along with the choice mothers made to focus on a target child with ASD, resulted in a skewed sample. Future studies should encourage study participation from all primary caregivers to acknowledge the variety of perspectives in one family. Studies should also include more diverse samples of children with ASD including children who identify as female. Another possible study limitation was the age of Emilia’s son (15 years old) as compared to the ages of the other participants’ sons at the time of the study (5–6 years old). It could have been possible that Emilia may not have recounted with accuracy the diagnostic circumstances of her oldest child. However, because Emilia’s report was quite detailed and it involved two diagnoses (one in Mexico and one in the US) in addition to her family immigrating to the US (a major event in any immigrant’s life), we have no reason to question the accuracy of her report.

Study findings demonstrated the heterogeneity that exists in understanding the ASD diagnosis process for immigrant, Mexican heritage families. A major contribution of this study is the detailed accounts of the process from ASD referral to diagnosis. Families identified timely treatment trajectories and specific barriers to diagnosis and treatment that have not yet been documented in the field. Future studies should use similar methodological tools to develop individualized diagnosis and treatment pathways that would support immigrant families who report similar barriers to diagnosis and treatment.

## Supplementary Information

Below is the link to the electronic supplementary material.

Supplementary file1 (DOCX 14 KB)

## Footnotes

1 All participants in this study were either born in Mexico and were living in the US (as either documented or undocumented citizens) at the time of the study, or their parents were from Mexico. Participants were either documented or undocumented. This study utilized the term “Mexican heritage” to refer to their national origin in reference to calls for researchers to include specific demographic information about race/ethnicity in their studies (Fuller & García Coll, 2010; MacMillan & Reschly, 1998).

2 Names of all participants and children are pseudonyms.

3 Name of all locations have been anonymized.
