# Supplementary material for: ASD Diagnosis and Treatment Experiences Among Mexican Heritage Families
Source: J Autism Dev Disord. 2022 Mar 19;53(3):1017–33. doi: 10.1007/s10803-022-05512-3 (PMC9986214; doi:10.1007/s10803-022-05512-3)
Supplement: Supplementary file 1 — Supplementary file1 (DOCX 14 KB) [file 10803_2022_5512_MOESM1_ESM.docx]

Supplementary Table

*Quoted Translations*

| **Spanish** | ***English*** |
| --- | --- |
| La persona que me lo miró ahí, él no quiso ni siquiera interactuar con la gente, él nada más se alejó, no quería que le hablaran, solamente jugaba. Entonces ella me dijo: "¿sabes qué? No te puedo dar un diagnóstico, pero lo voy a referir al Centro Regional. | *The person who saw him there, [my child] did not want to interact with the people, he just stayed away, he didn’t want them to talk to him, all he did was play. Therefore [the clinician] told me, ‘You know what? I cannot give you a diagnosis, but I am going to refer him to the Regional Center.”* |
| ...en tres años yo vi una gran diferencia...pero de los tres a los cuatro que empezó a cursar la escuela especial, fue cuando fue su avance más grande: los logros más grandes que él tuvo de hablar, de poderse expresar, de entender, de captar las cosas, pero fue la escuela la que me ayudó. | *...in three years I have seen a big difference...but from three to four when he started in special education, that's when he grew the most; the biggest gains he had were in speech, his ability to express himself, to comprehend, to understand things, but it was the school that helped me.* |
| ‘Yo los crié a ustedes y yo nunca tuve estos berrinches, es muy necio el niño y no se me hace normal esto". Entonces ella tuvo la experiencia de haber dicho: "Yo tuve bebés, los tuve a ustedes cuatro y con ninguno fue así, entonces hay algo | *‘I raised you all and [you] never had these tantrums, the boy is very strong willed and I do not think this is normal.’ So she had the experience to tell me: ‘I had babies, I had all four of you and none of you were like this, so something is up.’* |
| Y hacía mucho berrinche, y creo que cuando estaba haciendo el berrinche, lo querían controlar, él golpeaba a las personas que lo estaban sujetando. Ahí es donde también dijeron: "Es que le tienes que buscar ayuda". Y ahí es donde ellos me recomendaron a Pasitos, que es un lugar de centro de psicología en México para diagnósticos, y ahí es donde le diagnosticaron el autismo. | *And he was throwing a lot of tantrums, and I think that when he was throwing a tantrum, they tried to restrain him, he would hit the people that were trying to restrain him. That’s where they said: “You are going to have to find help.’ And that is where they recommended me to Pasitos, which is a psychology center here in Mexico for diagnosing, and that is where they diagnosed him with ASD.* |
| Todavía no teníamos una situación legal, no estábamos inmigrados. Gracias a Dios ahorita sí ya... Ahí es donde empezó la situación, porque nosotros no teníamos papeles. No le podían dar ayuda porque no teníamos MediCal, y yo no lo podía pagar. Lo único que yo podía hacer-- ellos me dijeron: ‘Lo único que puedes hacer es lo que la escuela te da.’ | *We still were not in a legal situation, we were not immigrated. Thank God now we are... That is where the situation started, because we were undocumented. They couldn’t help him because we didn’t have MediCal, and I couldn’t pay for it. All I could do-- they told me: ‘The only thing you can do is what the school gives you.’* |
| Entonces dije yo: "Cómo no me di cuenta de todos esos detalles. Si tan solo le hubiera dado ayuda desde chiquito." A Salvador le empecé la ayuda desde antes que empezara el año, yo empecé a empujar, "Es que quiero que me lo empiecen a diagnosticar, quiero que empiecen a tocar puertas, Hospital, Clínica. Quiero que me pongan en una lista de espera porque yo quiero que me lo detecten a tiempo porque no quiero que mi hijo pase lo que pasó Eric". Entonces de haberlo no creído, ahora me estaba adelantando…. | *So I said: ‘How did I not notice all of these details. If only I had given him help since he was younger.’ I began giving Salvador [youngest child] help before he turned one [year old], I began to push. ‘It’s because I want them to begin diagnosing him, I want them to start knocking on doors, Hospitals, Clinics. I want them to put me on a waiting list because I want him to be detected on time because I don’t want my son to go through what Eric [eldest child] did.’ Since believing [that my child had a problem], now I could anticipate things…* |
| Entonces, ya cuando me diagnosticaron a la niña, me empecé a fijar en Isaiah. Yo comenté aquí que me gustaría que vieran a Isaiah, porque veía algo de él también que no era normal, que no respondía cuando le hablaba, no volteaba, no balbuceaba nada... Era muy tranquilo, podía estarse acostado sin hacer nada ni llorar ni nada. Para mí no era normal. | *So, once they [The Regional Center] diagnosed my daughter, I began to notice Isaiah. I commented here that I would like them to see Isaiah, because I saw something in him too that wasn't normal, he did not respond when I talked to him, he didn't turn, he didn't babble at all… He was very calm, he could lay down and do nothing, not even cry nothing. For me it wasn't normal.* |
| Como en Isaiah yo lo descubrí antes, entró él en ese programa hasta los tres años, y a los tres años ya lo pusieron en las otras agencias, como Motiva… Después del diagnóstico, no fue mucho, porque fue nomás canalizarse a sus terapias de ABA. Es todo. | *Like with Isaiah I found out before, ...he entered that program until three years old, and at three years old they placed him with the other agencies, like Motiva… After the diagnosis, it wasn't a lot, because it was only orienting myself to his ABA therapies. That’s it.* |
| Yo pienso que a Isaiah le ayudó ABA, le ayudó la Motiva, le ayudó mucho. Él ya estaba aprendiendo a tratar de querer comunicar con sus ‘pets,’ y ahorita ya lo perdió, pero él ya trataba… O sea, ya era un buen avance porque él ya estaba relacionando, "Quiero algo, tengo que enseñar algo". Entonces ya perdió eso él ahora. Era la poca comunicación que tenía. En eso, aprendió a estar sentado. | *I think that ABA helped Isaiah, Motiva helped him, it helped him a lot. He was already learning how to try to communicate with his pets, and now he has lost that, but he was trying… I mean, it was a big development because he was beginning to understand, ‘I want something, I have to demonstrate something.’ So now he has lost that. That was the little bit of communication he had. Within that, he learned to sit.* |
| yo no he tenido comunicación de-- yo sí he venido a preguntar: ‘¿Y cuándo?’, ‘No, está en lista de espera’, ‘Okay.’ Bueno, ya me cansé de estarles preguntando, entonces también no me puedo sentar y estar esperando, porque podría no llegar. Entonces, yo ahorita como digo: ‘¿Qué hago?, ¿qué hago? El niño no habla, no avanza.’ Es difícil. | *I have not had communication about-- I have come to ask: ‘And when?’, ‘No, he is on the waiting list’, ‘Okay.’ Well, I am tired of asking them, but also I can’t just sit and wait, because it could not arrive. So right now I’m like: ‘What do I do? What do I do? He is not talking, not advancing, making gains.’ It is difficult.* |
| Y a esa enfermera yo ya la había conocido de hace muchos años. Y cuando miraba al niño que él se empezaba a golpear, que mordía y solamente con un jarro, ella me dijo: "No te vayas a molestar, pero yo pienso que tu niño tiene un autismo". Yo le dije: "No creo". Y yo pedí que cambiaran a esa enfermera por haberme dicho eso. Y luego, ya con las evaluaciones, con todos los estudios, era una de ir a Children Hospital tres veces por semana, cuatro veces por semana. Después, ya no era tanta la molestia; pero cuando me lo dijeron por primera vez, sí me molesté. Me molesté demasiado y cambiaron a la enfermera que venía a ver al niño y a trabajar conmigo. | *And I had already met that nurse many years ago. And when she saw my child hit himself, biting, and only with a jar, she said: ‘Don't be upset, but I think that your child has autism.’ I told her: ‘I don't believe that,” and I asked that they switch nurses because she told me that. And then, with the evaluations already, with all the studies, there was one where we had to go to Children’s Hospital three times a week, four times a week. Afterwards, I wasn’t as upset, but when they told me for the first time, I was upset. I got really upset and they changed the nurse that was coming to see my child and work with me.* |
| Y a esa enfermera yo ya la había conocido de hace muchos años. Y cuando miraba al niño que él se empezaba a golpear, que mordía y solamente con un jarro, ella me dijo: "No te vayas a molestar, pero yo pienso que tu niño tiene un autismo". Yo le dije: "No creo". Y yo pedí que cambiaran a esa enfermera por haberme dicho eso. Y luego, ya con las evaluaciones, con todos los estudios, era una de ir a Children Hospital tres veces por semana, cuatro veces por semana. Después, ya no era tanta la molestia; pero cuando me lo dijeron por primera vez, sí me molesté. Me molesté demasiado y cambiaron a la enfermera que venía a ver al niño y a trabajar conmigo. | *And I had already met that nurse many years ago. And when she saw my child hit himself, biting, and only with a jar, she said: ‘Don't be upset, but I think that your child has autism.’ I told her: ‘I don't believe that,” and I asked that they switch nurses because she told me that. And then, with the evaluations already, with all the studies, there was one where we had to go to Children’s Hospital three times a week, four times a week. Afterwards, I wasn’t as upset, but when they told me for the first time, I was upset. I got really upset and they changed the nurse that was coming to see my child and work with me.* |
| Ya era uno de que, "Okay. Él ya tiene los tres años, ya va a ir al Head Start". Head Start luego después me dice: "No, no, no". Me lo traían de un lado para otro. "Es que ya le corresponde al distrito porque ya es diferente.” El Centro Regional ya no me lo quería ver. Y batallé mucho hasta que me lo volvieron a evaluar, hicieron otra evaluación | *It was now, ‘Okay. He is three years old, he is going to Head Start.’ Then Head Start told me: ‘No, no, no.’ They had him going from one place to another. ‘It’s that now it’s the responsibility of the district because it’s different now.’ The Regional Center did not want to see him. And I fought a lot until they re-evaluated him, they gave him another evaluation.* |
